# “A Vegetarian vs. Conventional Hypocaloric Diet: The Effect on Physical Fitness in Response to Aerobic Exercise in Patients with Type 2 Diabetes.” A Parallel Randomized Study

**DOI:** 10.3390/nu8110671

**Published:** 2016-10-26

**Authors:** Jiri Veleba, Martin Matoulek, Martin Hill, Terezie Pelikanova, Hana Kahleova

**Affiliations:** 1 Institute for Clinical and Experimental Medicine, Videnska 1958/9, 14021 Prague, Czech Republic; jivb@ikem.cz (J.V.); tepe@ikem.cz (T.P.); 2 General University Hospital, 3rd Internal Clinic of Endocrinology and Metabolism, 12808 Prague, Czech Republic; mmato@vstj.cz; 3 Institute of Endocrinology, Narodni 8, 11394 Prague, Czech Republic; mhill@endo.cz

**Keywords:** insulin sensitivity, maximal oxygen consumption, maximal performance, physical fitness, type 2 diabetes, vegetarian diet

## Abstract

It has been shown that it is possible to modify macronutrient oxidation, physical fitness and resting energy expenditure (REE) by changes in diet composition. Furthermore, mitochondrial oxidation can be significantly increased by a diet with a low glycemic index. The purpose of our trial was to compare the effects of a vegetarian (V) and conventional diet (C) with the same caloric restriction (−500 kcal/day) on physical fitness and REE after 12 weeks of diet plus aerobic exercise in 74 patients with type 2 diabetes (T2D). An open, parallel, randomized study design was used. All meals were provided for the whole study duration. An individualized exercise program was prescribed to the participants and was conducted under supervision. Physical fitness was measured by spiroergometry and indirect calorimetry was performed at the start and after 12 weeks Repeated-measures ANOVA (Analysis of variance) models with between-subject (group) and within-subject (time) factors and interactions were used for evaluation of the relationships between continuous variables and factors. Maximal oxygen consumption (VO_2max_) increased by 12% in vegetarian group (V) (F = 13.1, *p* < 0.001, partial *η*^2^ = 0.171), whereas no significant change was observed in C (F = 0.7, *p* = 0.667; group × time F = 9.3, *p* = 0.004, partial *η*^2^ = 0.209). Maximal performance (Watt max) increased by 21% in V (F = 8.3, *p* < 0.001, partial *η*^2^ = 0.192), whereas it did not change in C (F = 1.0, *p* = 0.334; group × time F = 4.2, *p* = 0.048, partial *η*^2^ = 0.116). Our results indicate that V leads more effectively to improvement in physical fitness than C after aerobic exercise program.

## 1. Introduction

Dietary intervention and physical exercise are both cornerstones in the treatment of type 2 diabetes (T2D) patients [1]. A vegetarian diet is a promising way to reduce energy intake by consuming foods with a low energy density, with a fair degree of patient adherence [2,3].

The superior effects of a vegetarian diet on body weight, glycemic control, blood lipids, insulin sensitivity and oxidative stress markers compared with a conventional diet have been shown by us and others previously [2,3,4]. A vegetarian diet was also reported to reduce the content of intramuscular lipids [5].

Physical activity combats insulin resistance by several different mechanisms: by influencing changes in body composition such as reducing fat mass and volume of visceral fat and increasing fat-free mass, by enhancing insulin-stimulated glucose disposal in skeletal muscle, morphological changes in muscle, and by decreased glucose production in the liver [6]. To the best of our knowledge, a direct comparison between the effect of a vegetarian diet and a conventional hypocaloric diet on physical fitness and resting energy expenditure (REE) in subjects with T2D during aerobic exercise training has not yet been performed.

It has been shown that it is possible to modify macronutrient oxidation, physical fitness and resting energy expenditure (REE) by changes in diet composition. Furthermore, mitochondrial oxidation can be significantly increased by a diet with a low glycemic index. The aim of this secondary analysis was to compare the effects of vegetarian (V) and conventional diabetic diet (C) with the same caloric restriction (−500 kcal/day) on physical fitness and REE after 12 weeks of diet plus aerobic exercise in patients with type 2 diabetes (T2D).

## 2. Experimental Section

The characteristics of our study population and the methods can be found elsewhere [3]. Briefly: In the context of a randomized, open, parallel design, 74 patients with T2D treated by oral hypoglycemic agents, both men (47%) and women (53%) were randomly assigned either into the vegetarian group (V, *n* = 37) or the control group (C, *n* = 37) treated by conventional diet. Both diets were calorie-restricted (−500 kcal/day) according to the indirect calorimetry measurement [7]. The dietary interventions were combined with aerobic exercise for 12 weeks, performed under professional supervision. All meals were provided for the whole study duration. The study protocol was approved by the Institutional Ethics Committee of the Thomayer Hospital and Institute for Clinical and Experimental Medicine, Prague, Czech Republic. (The approval code is G-08-08-22.)

### 2.1. Diet

The vegetarian diet (~60% of energy from carbohydrates, 15% protein, and 25% fat) was based on whole plant foods (whole grains, legumes, vegetables, fruits and nuts). Animal products were rectricted to one portion of low-fat dairy a day. The composition of the conventional diabetic diet met the dietary guidelines of the Diabetes and Nutrition Study Group (DNSG) of the European Association for the Study of Diabetes (EASD) [8]. It derived 50% of energy from carbohydrates, 20% protein, less than 30% fat (≤7% energy from saturated fat, less than 200 mg/day of cholesterol/day).

### 2.2. Exercise

An individualized exercise program was prescribed to the participantsbased on previous physical activity and spiroergometry. Aerobic exercise was performed twice a week at 60% of maximal heart rate for 1 h under professional supervision at the sports center, and the third weekly session took place either at the sports center or at home. The participants used a sport-tester and a pedometer.

### 2.3. Medication

No changes in medication use were made, except for the case of repeated hypoglycemia (plasma glucose determined at the laboratory <4.4 mmol·L^−1^ or capillary glucose reading <3.4 mmol·L^−1^ accompanied by hypoglycemic symptoms). In this case, medications were reduced by a study physician following a standard protocol. All participants used an Accu-Chek Go glucometer (Roche, Basel, Switzerland).

### 2.4. Adherence

All visits to pick up meals were recorded. Three-day dietary records (two weekdays and one weekend day) were completed by each participant at baseline and at week 12, and analyzed by a registered dietician. High adherence was defined as the average daily energy intake being no more than 100 kcal in excess of the prescribed, medium adherence was less than 200 kcal in excess. Additional criteria for high adherence to vegetarian diet were the average daily cholesterol intake ≤50 mg, for medium adherence less than 100 mg. In the control group, the average daily cholesterol limit was ≤200 mg for high adherence, and less than 300 mg for medium adherence.

Adherence to the exercise program was defined as more than 75% of prescribed visits at the sports center (18/24).

### 2.5. Hunger and Depressive Symptoms

Hunger and depressive symptoms were assessed using the Three-Factor Eating Questionnaire [9] and the Beck Depression Inventory [10], respectively.

### 2.6. Statistical Analysis

The intention-to-treat analysis was used, and all participants were included. To eliminate skewed data distribution and heteroscedasticity, the original data was transformed to a Gaussian distribution before further processing by a power transformation using the statistical software Statgraphics Centurion, version XV from Statpoint Inc. (Herndon, Virginia, VA, USA). Non-homogeneities in the data were detected using residual analysis. Repeated-measures ANOVA (Analysis of variance) models with between-subject (group) and within-subject (time) factors and interactions were used. Factors of treatment group, subject and time were included in the model. Interactions between group and time (group × time) were calculated for each variable.

Paired *t*-tests were calculated within each group, to check the significance of changes from baseline. Bonferroni post-hoc correction for seven variables implies that *p*-values < 0.007 can be considered significant.

## 3. Results

**Figure 1:**
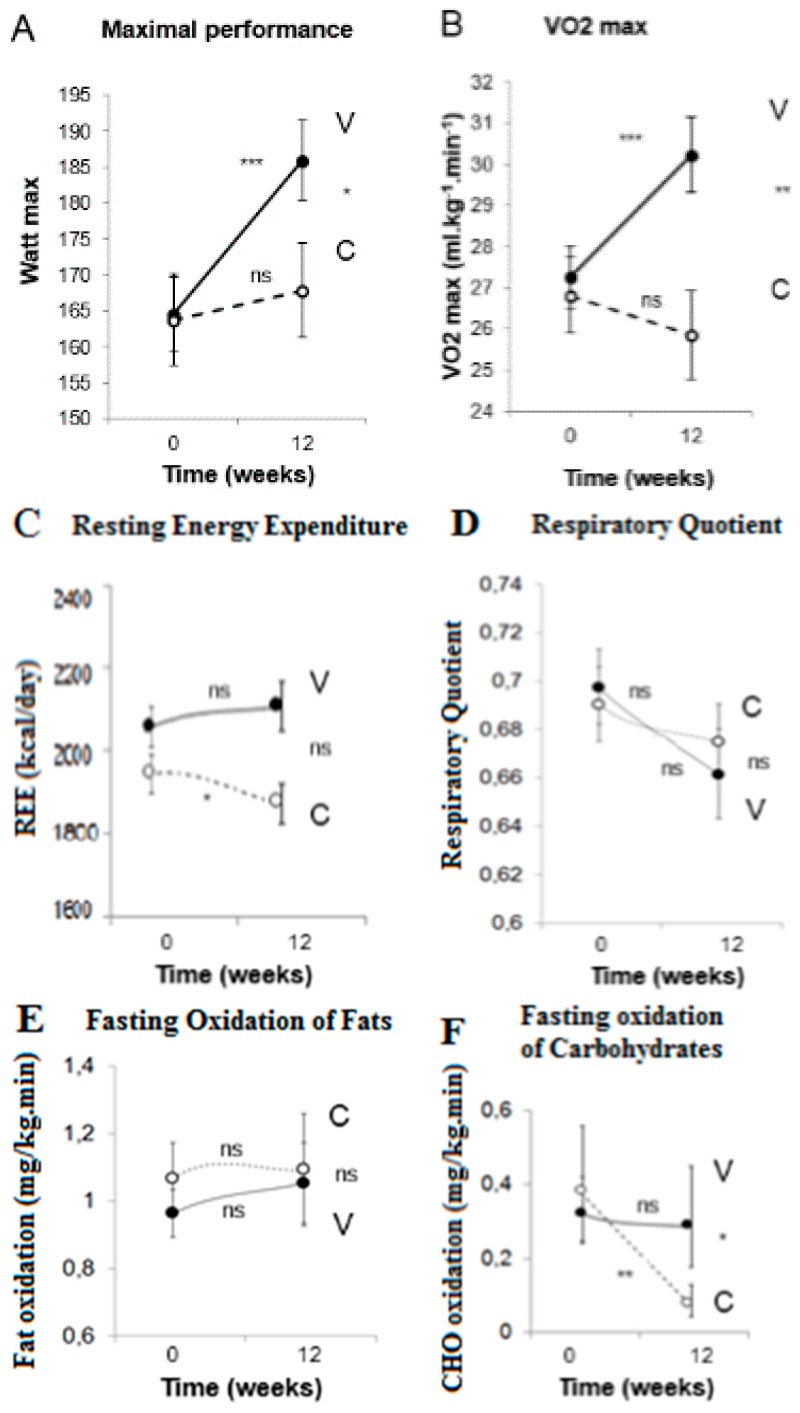

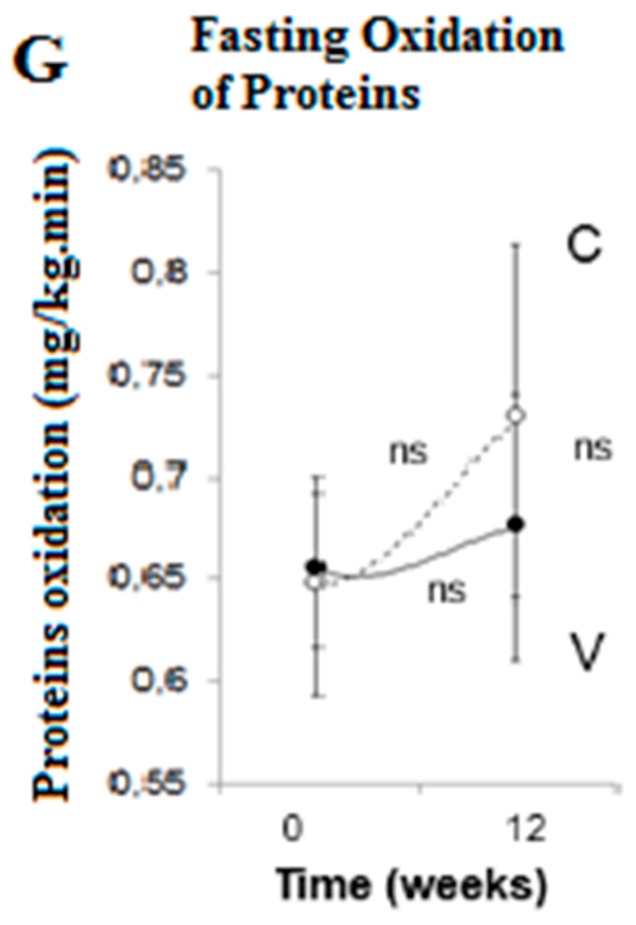
Changes in physical fitness in response to a vegetarian (V, full line and full circles) and conventional diet (C, dashed line and empty circles). Data are means ± 95% confidence intervals. Significant changes from baseline to 12 weeks within groups assessed by paired comparison *t*-tests are indicated by * for *p* < 0.05, ** for *p* < 0.01, and *** for *p* < 0.001. *p*-values for the interaction between factors group (vegetarian and control group) and time (0 and 12 weeks) assessed by repeated measures. ANOVA are: *p* = 0.048 for maximal performance (**A**), *p* = 0.004 for maximal oxygen consumption, VO_2max_ (**B**), *p* = 0.067 for resting energy expenditure (**C**), *p* = 0.666 for respiratory quotient (**D**), *p* = 0.793 for fasting oxidation of fats (**E**), *p* = 0.024 for fasting oxidation of carbohydrates (**F**), and *p* = 0.082 for fasting oxidation of protein (**G**).

Data are presented as means with 95% confidence intervals. Maximal performance (Watt max) increased by 21% in vegetarian group (V) (F = 8.3, *p* < 0.001, partial *η*^2^ = 0.192), whereas it did not change in control group (C) (F = 1.0, *p* = 0.334; group × time F = 4.2, *p* = 0.048, partial *η*^2^ = 0.116; Figure 1A). Maximal oxygen consumption (VO_2max_) increased by 12% in V (F = 13.1, *p* < 0.001, partial *η*^2^ = 0.171), whereas it did not change significantly in C (F = 0.7, *p* = 0.667; group × time F = 9.3 *p* = 0.004, partial *η*^2^ = 0.209; Figure 1B). REE remained constant in V (F = 0.6, *p* = 0.556), whereas it decreased in C (F = 4.1, *p* = 0.032, partial *η*^2^ = 0.113; group × time F = 2.8, *p* = 0.067; Figure 1C). The respiratory quotient did not change significantly in either group (F = 2.7, *p* = 0.0673 for V, and F = 1.5, *p* = 0.224 for C; group × time F = 0.4, *p* = 0.666; Figure 1D). No change in fasting oxidation of fat was observed in either group (F = 0.3, *p* = 0.742 for V, and F = 1.1, *p* = 0.318 for C; group × time F = 0.4, *p* = 0.658; Figure 1E). Fasting oxidation of carbohydrates decreased in C by 89% (F = 3.8, *p* = 0.01, partial *η*^2^ = 0.029), while it did not change in V (F = 0.2, *p* = 0.865; group × time F = 4.0, *p* = 0.024; partial *η*^2^ = 0.139; Figure 1F). Fasting oxidation of protein did not change in either group (F = 0.3, *p* = 0.742 for V, and F = 1.4, *p* = 0.259 for C; group × time F = 2.6, *p* = 0.082; Figure 1G).

### Adherence

The diet adherence was high among 55% of participants in V and 32% in C, medium among 22.5% in V and 39% in C, and low among 22.5% in V and 29% in C. Adherence to the exercise program was 90.3% in V and 80.6 in C.

## 4. Discussion

Our results show a slight improvement in physical fitness after a 12-week aerobic training program with a vegetarian diet compared with a conventional hypocaloric diet. David Nieman demonstrated in his review in 1999 [11] that vegetarianism and veganism do not diminish physical fitness. Several studies showed that endurance athletes and marathon runners might benefit from plant-based diets with an emphasis on high-carbohydrate and antioxidant-rich foods such as pasta, grains, cereals, legumes, vegetables, and dried fruits [12,13].

Numerous comparative studies reveal no fundamental differences in morphological or enzymatic equipment of skeletal muscle in vegetarians/vegans compared to omnivores [14,15]. In our study, we showed that visceral fat decreased more in V compared to C [3]. This might suggest the possible decrease of ectopic fat in the muscle, potentially related to improved physical fitness. Several studies reported lower levels of intramyocellular fat in vegetarians/vegans, implying their improved insulin sensitivity. However, the impact of this finding on physical fitness is unclear. Given the described athlete's paradox [6], where trained athletes have more intramyocellular fat than healthy subjects, and even more than those with type 2 diabetes, it is questionable if lower intramyocellular fat content can be expected to be related to better fitness.

Abete et al. showed that a diet with a lower glycemic index increases mitochondrial oxidation [16], which corresponds with our findings. Plant-based diets with an emphasis on whole plant foods have a low glycemic index due to the high fiber content [17]. In addition, it seems that participants in V were able to better utilize carbohydrates compared to the control group. Together with the increased insulin sensitivity demonstrated previously [3], these are markers of improved metabolic flexibility, which may partly explain the increased maximal performance and VO_2max_ in V.

Besides physiological mechanisms, we need to mention the potential role of psychological factors. We observed reduced hunger and a reduced Beck depressive score in V [18], pointing to a higher executive potential including a positive attitude toward exercise. This hypothesis is also supported by lower levels of leptin in V [3], which may potentiate readiness for physical activity through the central nervous system [19].

The strengths of the study are represented by the randomized, parallel design, providing all meals, and exercising under professional supervision. The study duration was reasonably long, allowing sufficient time for tracking the changes in response to the diet and exercise. However, the number of subjects and study duration preclude generalizing our study for free-living conditions. Further larger-scale, long-term studies are essential before offering recommendations in terms of vegetarian diet during aerobic exercise.

## 5. Conclusions

In conclusion, our results indicate that V leads more effectively to improvement in physical fitness than C after an aerobic exercise program. We have also observed a decrease in REE only in C in response to aerobic exercise. The lower glycemic index of V, the higher fasting oxidation of carbohydrates, and the possible increase in mitochondrial oxidation may be partly responsible for a trend toward greater REE with V after aerobic exercise. V might be a more convenient alternative in the nutritional treatment of T2D during an aerobic exercise program.
